# Supplementary material for: Greek Pig Farmers’ Perceptions and Experiences of Tail Biting and Tail Docking
Source: Animals (Basel). 2023 Feb 15;13(4):672. doi: 10.3390/ani13040672 (PMC9952019; doi:10.3390/ani13040672)
Supplement: Supplementary file 1 [file animals-13-00672-s001.zip › animals-2106023-supplementary.pdf]

Table S1. Studies on attitudes and perceptions of pig farmers towards tail biting.

Country	Main farming system	Year	Author	Main outcome
United Kingdom	Indoor and Outdoor production.Wide use of bedding	2019	Valros Anna and Barber Claire	The difference on the ranking of preventive measures between farmers and scientists
Ireland	Indoor production/Tail-docking/Slatted floors/ad libitum feeding/pelleted feed	2019	Haigh Amy and O'Driscoll Keelin	Stocking densities and food issues are recognized by farmers
Finland	Indoor production/Intact tails/Wide use of bedding/Liquid feeding	2016	Valros Anna et al.	Ranking of preventive measures varies between farmers due to different experiences of tail docking
Sweden	Indoor production/Intact tails/Wide use of bedding	2016	Wallgren Torun et al.	Straw access can prevent tail biting without serious problems for the manure systems
Netherlands	Indoor production/Tail-docking/Slatted floors/ad libitum feeding	2013	Bracke M.B.M. et al.	Climate recognized as the main risk factor in contrast to enrichment
United Kingdom	Indoor and Outdoor production.Wide use of bedding	2001	Hunter E.J. et al.	Removing the bitten pig is the most common action dealing with an outbreak while tail docking is the most common practice preventing it.

Table S2. The farmer intervenes when he sees :.

Biting marks on the tail
Fresh blood on the tail
Missing part of the tail
One bitten animal in the cell
More than one bitten animal in the cell

Table S3. Intervention measures used by Greek farmers in case of a tail-biting outbreak. Scale: 1 (Doesn't help at all) to 5 (It helps a lot).

Intervention measure	N	Mean	(±stdv)
Remove Victim	76	4.37	1.220
Remove biter	73	3.73	1.592
Adjust ventilation	68	3.13	1.573
Adjust temperature	67	3.09	1.621
Reduce stocking density	71	2.85	1.582
Adding enrichment	68	2.68	1.606
Antibiotics to victims	72	2.54	1.669
Anti-biting substance	71	2.32	1.510
Reducing light	62	1.84	1.296
Pig lick blocks	54	1.81	1.347

Table S4. Opinions of pig farmers on the importance of risk factors for tail biting given in the questionnaire. Scale: 1 (Not important at all) to 5 (Very important).

Risk Factors	N	Mean	(±stdv)
Feed quality (over-under supply of minerals)	77	3.75	1.387
Stocking density	78	3.58	1.559
Ventilation	79	3.44	1.500
Tail length	78	3.35	1.650
High temperatures	78	3.04	1.481
Poor health	78	2.99	1.608
Lack of stable microclimate	75	2.96	1.493
High humidity	76	2.95	1.469
Piglets mixing	77	2.87	1.665
Intense weather fluctuations	76	2.53	1.501
Lack of appropriate enrichment material	76	2.50	1.428
Number of water nipples in pen	77	2.49	1.475
Pen hygiene/cleanness	75	2.47	1.398
Number of feeders in pen	77	2.45	1.447
Feeding always at the same time of day	72	2.38	1.614
Runts or decreased growth	75	2.33	1.519
Caretakers bad handling	75	2.33	1.501
High contrast due to light coming from windows	75	1.71	1.171
Breed of pigs	72	1.69	1.229
Sex of pigs	72	1.50	0.839
